# Supplementary material for: WordCommentsAnalyzer: A windows software tool for qualitative research
Source: F1000Res. 2018 Sep 4;7:536. Originally published 2018 May 3. [Version 2] doi: 10.12688/f1000research.14819.2 (PMC6137411; doi:10.12688/f1000research.14819.2)
Supplement: Supplementary file 3 [file f1000research-7-17614-s0002.tgz › b2a79496-4ada-47c4-9682-a99ce867836d.zip!/Eight Iranian Health Journals/Journal of Health System Research _12.docx]

© Journal of Health System Research . This work is licensed under CC BY-NC 4.0 (https://creativecommons.org/licenses/by-nc/4.0/).

Volume & Issue: دوره 11، شماره 2، 1394

Title: افزایش پرداخت سازمان های بیمه برای خدمات تشخیصی تصویربرداری با رزونانس مغناطیسی (MRI) و عوامل مؤثر در آن : مطالعه مروری

http://hsr.mui.ac.ir/index.php/jhsr/article/view/1652

مقدمه: استفاده از خدمات تشخیصی پر هزینه با تکنولوژی سطح بالا در دهه‌ی گذشته افزایش یافته است. تحقیقاتی که انجام شده است دلایل متعددی را با توجه به سیستم بهداشت درمانی موجود در هر کشور گزارش کرده‌اند ، ازجمله مسن شدن جمعیت ،پیشرفت در تکنولوژی‌ها‌ی تشخیصی، افزایش تعداد رادیولوژیست‌هاو غیره. لذا توجه به اقتصاد بهداشت این خدمت روز به روز در حال افزایش است. روش‌ها: این مطالعه یک مطالعه مروری است که با استفاده از بانک‌های اطلاعاتی ISIو Science Direct , BMC , Clinical Imaging, Journal of Health Economics و دیگر مجلات مرتبط در جستجوی شناخت عوامل مؤثر در افزایش استفاده از خدمات تشخیصی(MRI یا Magnetic resonance imaging) در سال‌های اخیر با توجه به مطالعات خارجی و داخلی می‌باشد. یافته‌ها: میزان استفاده از خدمات MRI افزایش یافته است. این افزایش به دلایل متعددی می‌باشد . در مطالعه حاضر علل افزایش هزینه‌های خدمات تشخیصی در 5 دسته قرار گرفت، شامل: عوامل تکنولوژیکی، عوامل مربوط به پزشکان ، عوامل مربوط به بیمار و جامعه ، عوامل مربوط به رادیولوژیست‌ها و عوامل مربوط به کارکرد بازار نظام سلامت. این طبقات هر کدام جزئیات و زیرطبقه‌های مربوط به خود را دارند که باید متناسب با شرایط هر کشور جهت سیاست‌گذاری تعریف شوند. نتیجه‌گیری: فرهنگ کاربرد خدمات تشخیصی باید هم در جوامع پزشکی (پزشکان ، رادیولوژیست‌ها و......) ، هم برای سیاست‌گذاری و هم بیماران و جامعه ارتقا یابد. سیاست‌گذاری‌های غلط در کشورهای جهان سوم سهم عمده‌ای از درآمد آن‌ها را کسر که ناشی از عدم کارشناسی دقیق در خصوص اثربخشی و کارایی این نوع خدمات است. فرهنگ‌سازی برای جامعه و تأکید بر جنبه‌های مختلف قانونی، آموزشی و فرهنگی برای پزشکان تدابیر لازم را نیاز دارد.

Title: ارتباط حمایت اجتماعی درك شده با مشخصات دموگرافیک دربیماران مبتلا به سرطان تحت شیمی درمانی

http://hsr.mui.ac.ir/index.php/jhsr/article/view/1469

چكيده   مقدمه: تشخیص سرطان تجربه‌ای بسیار ناخوشایند و غیرقابل باور برای هر فرد است که دامنه‌اي از علائم شامل درد و انواع ناراحتي‌هاي جسمي و رواني‌را به همراه دارد. حمايت اجتماعي يک منبع قابل اعتماد براي کمک به بيماران سرطاني در سازگاري با بیماری و علائم آن است که کيفيت زندگي را افزايش مي‎دهد. این مطالعه به منظور بررسی حمايت اجتماعي درک شده و ارتباط آن با برخی مشخصات دموگرافیک در بیماران مبتلا به سرطان انجام گردید. روش‌ها: این مطالعه توصیفی تحلیلی در سال1392 در میان 117 نفر از بيماران مبتلا به سرطان تحت شیمی‌درمانی در بیمارستان سیدالشهدا اصفهان که به صورت نمونه‌گیری مستمر انتخاب شدند، انجام گردید. اطلاعات با استفاده از پرسش‌نامه‌اي شامل دو قسمت خصوصيات دموگرافيك و حمایت اجتماعی جمع‌آوری و با آزمون‌های آماری توصیفی و تحلیلی تجزیه و تحلیل شد. یافته‌ها: حمایت اجتماعی مشارکت‌کنندگان با میانگین (42/9SD=)21/59 در دو سطح پایین (3/57٪) و متوسط (7/42 ٪) قرار داشت. ارتباط حمایت اجتماعی با سن معکوس (02/0P=) و با سطح تحصیلات مستقیم (00/0P=) بود. میانگین حمایت اجتماعی در افراد مجرد بیش از افراد متأهل و افراد مطلقه و بیوه (02/0P=) و در زنان بیشتر از مردان بود (01/0P=). نتیجه‌گیری: یافته‌ها حاکی از پایین بودن سطح حمایت اجتماعی در افراد بود. هم‌چنین بر اساس نتایج مردان، افراد با سطح تحصیلات پایین‌تر و افراد بیوه و مطلقه حمایت اجتماعی کمتری دریافت نموده بودند و این حمایت با افزایش سن نیز کاهش می یافت. این مسأله توجه بیشتر به حمایت اجتماعی در این بیماران با‎لاخص در گروه‌های با سطح حمایت پایین‌تر را می‎طلبد.

Title: تأثیر آموزش بر آگاهی و نگرش دانش‌آموزان شهر ایذه در مورد بیماری‌های قلب و عروق: کاربرد مدل اعتقاد بهداشتی

http://hsr.mui.ac.ir/index.php/jhsr/article/view/1615

مقدمه: بررسي‌هاي موجود حاكي از اين هستند كه اغلب رفتارهاي بهداشتي و غير بهداشتي افراد در سنين نوجواني شكل گرفته و تداوم مي‌يابد و آموزش‌پذيري از خصيصه اين سنين است. بنابراين سرمايه‌گذاري در امر سلامت و آموزش جوانان درجهت پیشگیری از بيماري‌های قلبی عروقی، امري زير بنايي تلقي مي‌شود. این پژوهش به منظور بررسی تأثیر آموزش بر اساس سازه‌های مدل اعتقاد بهداشتی بر آگاهی و نگرش دانش‌آموزان در مورد بیماری‌های قلبی عروقی صورت گرفت. روش‌ها: در اين مطالعه نيمه‌تجربي(Quasi- Experimental)  از نوع قبل و بعد، از ميان كل دانش‌آموزان دبيرستاني ساکن شهر ايذه 196 نفر (79 دختر و 117 پسر) به شيوه نمونه‌برداری تصادفی چند مرحله‌ای انتخاب شدند. پرسش‌نامه خودساخته بر اساس اهداف تهيه و پس از تأييد روايي و پايايي آن در دو مرحله قبل و بعد از مداخله تكميل شد. داده‌ها با استفاده از نرم‌افزر SPSS20 ،آمار توصيفي و آزمون‌هاي آماري t زوجی، همبستگي پيرسون و اسپيرمن در سطح 05/0= α تجزيه و تحليل شدند. یافته‌ها: ميانگين نمره آگاهي دانش‌آموزان در زمينه عوامل خطرساز بيماري‌هاي قلب و عروق قبل از آموزش 18/4±95/12 بود كه بعد از آموزش به 48/4±85/23 افزايش يافت (001/0>p). ميانگين نمره نگرش نيز بعد از آموزش از 21/9±10/68 به  27/8±44/82 افزايش يافت (001/0>p). ميانگين نمره كليه سازه‌هاي مدل (حساسيت درك شده،شدت درك شده، منافع و موانع درك شده،خودكارآمدي درك شده و راهنماي عمل) در قبل و بعد از مداخله آموزشي اختلاف معنی‌داري داشت (001/0>p). نتیجه‌گیری: نتايج این مطالعه اثربخشی مداخله آموزشی بر اساس الگوي اعتقاد بهداشتی را در جهت ارتقای سطح آگاهی و تأثیر بر سازه هاي حساسیت، شدت، منافع درک شده، موانع و خود کارآمدی درک شده و راهنماي عمل دانش آموزان در رابطه با بيماري‌های قلبی و روش‌هاي پيشگيري از آن را تأیید می‌نماید، لذا جهت ارتقاي رفتارهاي محافظت‌کننده در برابر بیماری‌های قلبی عروقی و در نهایت پیشگیري از ابتلا به این بیماری‌ها در سنین بالاتر، به کارگیري برنامه‌ي آموزشی بر اساس این مدل به عنوان يك اولويت آموزشي در مدارس مي‌بايست مورد توجه مسؤولين و مديران بهداشتي و آموزشي كشور قرار گيرد.

Title: بررسی تأثیر استفاده از سایلنسر جذبی در کاهش صدای فرکانس پایین فن محوری ایرانی

http://hsr.mui.ac.ir/index.php/jhsr/article/view/1759

مقدمه: سیستم‎های تهویه ازجمله مهم‎ترین منابع صدا در ساختمان‎ها هستند که از بخش‎های چون فن، کانال یا توزیع‌کننده‎ها، توزیع و به محیط اطراف انتشار می‎یابند. فن‎های محوری در صنعت بسیار استفاده می‎شوند و سایلنسرها از جمله راه‌های کنترل صدای فن ها محسوب می‎شوند. هدف از این مطالعه بررسی تأثیر استفاده از سایلنسر جذبی در کاهش صدای فرکانس پایین فن محوری ایرانی است. روش‌ها: در این مطالعه از کانال گالوانیزه 6/. میلی‌متر با ابعاد 30*30 سانتی متر مربع و فن محوری استفاده شد. سایلنسر جذبی نیز از جنس کانال به گونه‌ای طراحی شد تا سطح مقطع کانال و سایلنسر پس از تعبیه جاذب در کانال باهم یکسان شود. سایلنسر جذبی به طول 50 سانتی‌متر و در آن از جاذب پشم معدنی (دانسیته Kg/m3  80 و 100  و ضخامت   cm5 و 10 ) استفاده شده است. برای تعیین تراز فشار صوت فن در کانال، دو میکروفون تعبیه شده است که با استفاده از نرم‌افزار MATLAB و در پهنای باند یک سوم اکتاو باند و در دورهای متغیر از 600-2500 دور بر دقیقه صورت گرفته شده است. در این مطالعه به بررسی تعدادی از متغیرهایی که بر عملکرد سایلنسر جذبی در کانال مؤثر است پرداخته شده است و تأثیر آن‌ها در کاهش تراز فشار صوت سایلنسر مورد بررسی قرار گرفته شده است. یافته‌ها: حداکثر 2 دسی بل کاهش فشار صوت در فرکانس‌های پایین توسط سایلنسر جذبی طراحی شده که هم سطح با کانال طراحی شده، به دست آمده است. در این مطالعه با افزایش میزان ضخامت در ماده جاذب سایلنسر جذبی تغییر چندانی در کاهش صدا وجود نداشت اما با افزایش چگالی از  Kg/m380 به 100نتایج بهتری در کاهش تراز فشار صوت دیده شد و باعث افزایش میزان میزان کاهش تراز فشارصوت (NR) شد. نتیجه‌گیری: با توجه به بررسی داده‎های حاصل از مطالعه مذکور، می‎توان در سیستم‎های تهویه از سایلنسر با ضخامت 5 سانتی متر با دانسیته Kg/m3 100 در کاهش فشار صوت در فرکانس پایین‌تر از 250 هرتز به عنوان وسیله‎ای مؤثر استفاده کرد.

Title: بررسی رابطه خودکارآمدی و هوش هیجانی با انگیزه پیشرفت در دانشجویان

http://hsr.mui.ac.ir/index.php/jhsr/article/view/1803

مقدمه: با توجه به اهمیت و نقش نیروهای کارآمد و توانمند در کشور و رسالت مهم دانشگاه در پرورش استعدادهای دانشجویان و نظر به این‌‌‌که در ایجاد انگیزه پیشرفت عوامل درون داد شامل هوش هیجانی و خودکارآمدی نقش به‌سزایی دارند لذا مطالعه حاضر با هدف بررسی رابطه خودکارآمدی و هوش هیجانی (Emotional Intelligence) با انگیزه پیشرفت در دانشجویان پیام نور انجام گرفت. روش‌ها: در این مطالعه توصیفی-تحلیلی از نوع همبستگی، 341 نفر از دانشجویان دانشگاه پیام نور بیرجند که به روش نمونه‌گیری طبقه‌ای تصادفی انتخاب شدند، مورد بررسی قرار گرفتند. ابزار پژوهش شامل سه پرسش‌نامه هوش هیجانی شرینگ، خودکارآمدی عمومی شرر و انگیزه پیشرفت تحصیلی هرمنس بود. داده‌ها با استفاده از نرم افزار 15  SPSSو آزمون‌های آماری T مستقل، آنالیزواریانس یک‌طرفه، ضریب همبستگی پیرسون و رگرسیون چندمتغیره در سطح 05/0 مورد تجزیه و تحلیل قرار گرفت. یافته‌ها: از میان دانشجویان مورد مطالعه 174 نفر (51 % )مذکر و 262 نفر (7/76%) مجرد بودند. نتايج ضریب همبستگی پیرسون نشان داد رابطه مثبت و معنی‌داری بین خودکارآمدی و نیز مؤلفه‌های خودآگاهی و مهارت اجتماعی با انگیزه پیشرفت تحصیلی وجود دارد. میانگین نمره انگیزه پیشرفت تحصیلی در دانشجویان مؤنث نسبت به مذکر و میانگین نمره خودکارآمدی در دانشجویان مذکر نسبت به مؤنث بیشتر است؛ ولی از نظر هوش هیجانی در دو گروه تفاوتی مشاهده نشد. هم‌چنین در هیچ‌کدام از متغیرهای مذکور در رده‌های مختلف سنی و نیز رشته های مختلف تحصیلی تفاوتی به دست نیامد. نتیجه‌گیری: نظر به این‌که همبستگی مثبت و معنی‌داری بین خودکارآمدی، خودآگاهی و مهارت اجتماعی با انگیزه‌ی پیشرفت تحصیلی دانشجویان مشاهده گردید لذا پیشنهاد می‌شود که با افزایش خودکارآمدی و هوش هیجانی از طریق اعمال روش‌های مناسب و غنی کردن محیط پرورشی، انگیزه‌ی پیشرفت تحصیلی دانش‌جویان را ارتقا دهیم.

Title: توزیع مقدار فعالیت بدنی بر اساس جنسیت و شاخص توده‌ی بدنی نسبی در دانش‌آموزان دارای اضافه وزن و چاق

http://hsr.mui.ac.ir/index.php/jhsr/article/view/1843

مقدمه: مطالعات زیادی نشان می‌دهند که افزایش شاخص توده بدنی نسبی (RBMI یا Relative Body Mass Index) ، کاهش فعالیت بدنی را به دنبال خواهد داشت. هدف این مطالعه بررسی توزیع مقدار فعالیت بدنی بر اساس شاخص جنسیت و توده‌ی بدنی نسبی در دانش‌آموزان دارای اضافه وزن و چاق می‌باشد. روش‌ها: این مطالعه توصیفی مقطعی به منظور بررسی توزیع مقدار فعالیت بدنی بر اساس شاخص جنسیت و توده‌ی بدنی نسبی در دانش آموزان دارای اضافه وزن و چاق در سال 1392 انجام گردید. برای این منظور تعداد 300 دانش آموز پایه ی پنجم و ششم ابتدایی به روش تصادفی از بین مدارس نواحی مختلف آموزش و پرورش استان قزوین انتخاب شدند. پرونده ی بهداشتی آنان بررسی شد و آنهاییکه بر اساس الگوی مرکز آمار بهداشتی (NCHS یا Statistics National Center for Health) دارای شاخص توده ی بدنی بالای 85 بودند انتخاب شدند. فعالیت بدنی واحدهای پژوهش با استفاده از یک پرسش‌نامه مناسب و مشابه (IPAQ-SF یا International Physical Activity Questionnaire- Short Form) مورد بررسی قرار گرفت. داده‌ها با روش‌های مناسب آماری در نرم‌افزار SPSS ویرایش 17 مورد تجزیه و تحلیل قرار گرفت. یافته‌ها: تجزیه و تحلیل داده‌ها نشان داد که فراوانی چاقی و اضافه وزن در دو جنس تفاوت معنی‌داری دارد (001/0 P<). دانش‌آموزان دارای اضافه وزن بدون توجه به جنسیتی که دارند، دارای فعالیت بدنی بیشتری نسبت به دانش‌آموزان چاق بوده و میانگین شاخص‌های MPA, VPA و MET نیز بر اساس توزیع جنسیتی و شاخص توده‌ی بدنی نسبی تفادت معنی‌داری دارند (001/0 P<). نتیجه‌گیری: از آن‌جایی‌که افزایش شاخص توده بدنی نسبی باعث کاهش فعالیت بدنی دانش‌آموزان هر دو جنس می‌گردد، لذا مهم است که با تهیه و تدوین برنامه‌های مناسبی باعث افزایش فعالیت بدنی بیشتر در دانش‌آموزان چاق به نسبت دانش‌آموزان دارای اضافه وزن گردیم.

Title: ارتباط بین سازه های مدل اعتقاد بهداشتی با رفتارهای پیشگیری کننده از ایدز در معتادین

http://hsr.mui.ac.ir/index.php/jhsr/article/view/1969

مقدمه: ایدز، یکی از مشکلات بهداشتی در جهان به ویژه در کشورهای در حال توسعه است و معتادین به مواد مخدر تقریبا 15/68٪ موارد ایدز در ایران را تشکیل داده، عامل اصلی گسترش ایدز محسوب می‌شوند. هدف این مطالعه تعیین ارتباط بین سازه‌های مدل اعتقاد بهداشتی با رفتارهای پیشگیری‌کننده از ایدز در معتادین خرم‌آباد می‌باشد. روش‌ها: پژوهش حاضر یک مطالعه توصیفی- تحلیلی از نوع مقطعی است که بر روی 88 نفر از معتادینی که در مرکز بازتوانی خرم آباد نگهداری می‌شدند، در سال 1392-1391 انجام شد. نمونه‌ها به صورت سرشماری وارد مطالعه شدند. ابزار گردآوری اطلاعات، پرسش‌نامه‌ای مشتمل بر سازه‌های مدل اعتقاد بهداشتی، آگاهی و رفتارهای پیشگیری‌کننده از ایدز بود. داده ها با آزمون‌های آماری آنالیز رگرسیون خطی چندگانه و ضریب همبستگی پیرسون تجزیه وتحلیل گردید. یافته‌ها: سازههاي مدل اعتقاد بهداشتی به همراه آگاهی در مجموع 46% واریانس رفتارهاي پیشگیري‌کننده از ایدز را توضیح دادند. در بین این متغیرها، میزان پیشگویی‌کنندگی متغیر خودکارآمدی درک شده (39/0ß=) بیش ازسایرمتغیرها بود و متغیر آگاهی (21/0ß=) در مرتبه‌ی بعدی بود. نتیجه‌گیری: یافته‌های پژوهش حاضر، رابطه بین خودکارآمدی، شدت و موانع درک شده را در اتخاذ رفتارهای پیشگیری‌کننده از ایدز تأیید می‌کند. نتایج این مطالعه می‌تواند در استراتژی‌های مداخله‌ای تئوری محور، به منظور ایجاد و تغییر رفتار بهداشتی مورد استفاده قرار گیرد.

Title: رابطه استرس شغلی و سابقه حوادث ترافیکی با نوبت کاری در رانندگان آژانس های درون شهری یزد

http://hsr.mui.ac.ir/index.php/jhsr/article/view/1970

مقدمه: با توجه به پیامدهای استرس شغلی بر روی رانندگان و هم‌چنین حوادث ترافیکی که سبب مرگ و میر و خسارت خواهد شد، لذا این مطالعه با هدف بررسی ارتباط استرس شغلی و سابقه حادثه در رانندگان آژانس‌های درون شهری یزد در دو گروه نوبت کار و غیر نوبت کار انجام گردید. روش‌ها: مطالعه حاضر یک مطالعه پژوهشی تحلیلی است که به روش موردی- شاهدی در سال 1392 و در رانندگان مربوط به 10 آژانس درون شهری یزد انجام گرفت. به همین منظور، تعداد 142 راننده نوبت کار و 125 نفر غیر نوبت کار به روش تصادفی- سهمیه‌ای انتخاب گردیدند. به منظور گردآوری داده‌های پژوهش از پرسش‌نامه فیلیپ ال رایس (Phillip L. Rice) استفاده شد. سپس نتایج توسط نرم‌افزار spss20 مورد تجزیه و تحلیل قرار گرفتند. یافته‌ها: آنالیز داده‌ها نشان داد که درصد نوبت کاران دارای سابقه حادثه (4/75٪) بسیار بیشتر از غیر نوبت کاران (56٪) است. هم‌چنین میانگین نمره استرس بر حسب سابقه نیز در نوبت کاران (61/26±74/169) بالاتر از غیر نوبت کاران (05/28±47/160) می‌باشد که این اختلاف معنی‌دار (02/0P= ) است. نتیجه‌گیری: نتایج به دست آمده حاکی از آن است که نوبت کاری باعث افزایش استرس شغلی رانندگان شده است و علاوه بر کاهش عملکرد فرد، سبب ایجاد حوادث نیز می‌گردد. لذا لازم است ضمن بررسى جامع‌تر ميزان استرس در رانندگان، تدابير لازم جهت كاهش پيامدهاى آن نیز انديشيده شود.

Title: تبيين تجارب استرس و استراتژي‌هاي سازگاری در معلمان زن مقطع ابتدایی

http://hsr.mui.ac.ir/index.php/jhsr/article/view/1977

مقدمه: شناسایی و درک عوامل اثرگذار بر بروز استرس معلمان زن مقطع ابتدایی و نحوه سازگاری با این عوامل می‌تواند موجب تغییرات شگرف در توسعه نظام آموزشی کشور شده و زمينه‌اي براي طراحی مداخلات آموزشی مناسب در راستاي ارتقاء سلامت افراد و جوامع باشد. هدف از این مطالعه تبيين تجارب استرس و استراتژي‌هاي سازگاری معلمان زن مقطع ابتدایی از ديدگاه آنان بود. روش‌ها: این مطالعه کیفی با مشارکت 46 نفر از معلمان زن مقطع ابتدایی شاغل در مدارس شهری و روستایی استان لرستان با نمونه‌گيري  تركيبي مبتني بر هدف به روش بحث متمركز (Focus group) به صورت نیمه‌ساختار یافته (Semi structured) در گروه‌های پنج تا ده نفره در سال 1392 طراحي و اجرا شد. پس از کسب رضایت‌نامه كتبي، داده‌ها جمع‌آوری، ضبط، دستنویسی و پس از کنترل صحت ثبت داده‌ها، با روش تحليل محتوي (Content Analysis) مورد تجزیه و تحلیل قرار گرفت. جهت حمایت از صحت و استحکام داده‌ها معیار مقبولیت، تأییدپذیری و انتقال‌پذیری لحاظ گردید. یافته‌ها: پنج مضمون اصلی شامل ماهيت استرس‌زایی شغل، مهندسی مجدد سازمانی، عوامل فرهنگی- اجتماعی، عوامل مربوط به فرد و تعارض نقش‌ها استخراج شد. نتیجه‌گیری: شناخت موضوعات در زمينه استرس و استراتژي‌هاي سازگاری می‌تواند در حل مشكلات آموزشی اثرگذاربوده و افقي جديد در انجام تحقیقات جامع‌تر برای محققان فراهم نمايد. به نظر می‌رسد استفاده از مدل‌های اجتماعی و مدل‌های در طراحی فرآیندهايی مانند پایش و نظارت‌ها، سیستم‌های آموزش معلمان، ارزش‌گذاری و تغییر در فرآیند ارتقاء شغلی مؤثر باشد.

Title: غلظت ویتامین D سرم با شاخص‌های سندرم متابولیک در زنان

http://hsr.mui.ac.ir/index.php/jhsr/article/view/1998

مقدمه: مقدمه: برخی مطالعات مقطعی ارتباط کمبود ویتامین D با اجزای سندرم متابولیک را نشان داده‌اند، اما یافته‌های فعلی کاملاً با یکدیگر هماهنگ نیستند. با توجه به شیوع بالای کمبود ویتامین D در ایران، مطالعه حاضر در راستای این هدف طراحی گردید. روش‌ها: 156 زن بالای 30 سال مبتلا به سندرم متابولیک به روش نمونه‌گیری تصادفی متوالی وارد مطالعه شدند. غلظت 25 هیدروکسی ویتامین D سرم [25(OH)D] و اجزای سندرم متابولیک اندازه‌گیری شد. سندرم متابولیک بر اساس (NCEP:ATP-III یا National Cholesterol Education Program/Adult Treatment Panel III) تعریف شد. افراد بر اساس غلظت 25(OH)D سرم به سه گروه طبقه‌بندی شدند. یافته‌ها: میانگین غلظت‌های 25(OH)D سرم 8/10 ± 5/20 نانوگرم در میلی‌لیتر مشاهده شد. 5/54 % زنان کمبود ویتامین D، 1/23% در معرض خطر کمبود و 4/22% دارای سطوح نرمال ویتامین D بودند. بعد از تعدیل سن، BMI و فعالیت فیزیکی، آنالیز ANCOVA ارتباط آماری معکوس و معنی‌داری بین غلظت 25(OH)D سرم و قند خون ناشتا نشان داد (003/0P =). آنالیز MANCOVA ارتباط مستقیم و معنی‌داری بین غلظت 25(OH)D با سطوح HDL-C سرم نشان داد (14 0/0P = ). ارتباط دور کمر و غلظت 25(OH)D از نظر آماری معنی‌دار نبود. ارتباط آماری معنی‌داری بین غلظت 25(OH)D با سایر اجزای سندرم متابولیک مشاهده نشد. نتیجه‌گیری: ارتباط 25(OH)D سرم با قند خون ناشتا و HDL-C می‌تواند پیش‌گوکننده بیماری‌های مزمن باشد، اما برای تعیین نقش ویتامین D با سندرم متابولیک و بیماری‌های مرتبط با آن طراحی مطالعات آینده‌نگر و کارآزمایی‌های بالینی ضروری به نظر می‌رسد.

Title: ارزیابی تراز صداي محيطي و میزان مواجهه با صدا در يك صنعت فولاد

http://hsr.mui.ac.ir/index.php/jhsr/article/view/2038

مقدمه: در صنعت فولاد گستره وسیعی از تجهیزات و ماشین آلات در فرآیند تولید مورد استفاده قرار می‎گیرند که از منابع صدای آزاردهنده محسوب می‎گردند. هدف این مطالعه ارزيابي تراز صداي محيطي و میزان مواجهه‌ با صدا در واحدهای مختلف يك صنعت فولاد بود. روش‌ها: پس از شناسایی منابع و ویژگی‎های صوتی تجهیزات در فرایند تولید، اندازه‎گیری تراز صدای محیطی و میزان مواجهه‌ فردي به ترتیب با استفاده از ترازسنج صدا مدلCell.450 بر طبق استاندارد 9612 ISO  و دوزیمتر مدل TES-1345انجام گردید. توزیع تراز صدا در سطح کارگاه‌ها به‌صورت نقشه صوتی با استفاده از نرم‌افزار AutoCAD تهیه گردید. ميزان افت شنوايي كارگران درطول سه سال متوالی از پرونده كارگران استخراج و نسبت به تراز صدای معادل مواجهه و دز صدای دریافتی مورد آنالیز قرار گرفت. یافته‌ها: در كل کارخانه از مجموع ايستگاه‌هاي اندازه‌گيري 5/18% در محدوده خطر قرار داشت. متوسط تراز فشار صوت در 4 واحد اصلی کوره، انرژی، چدنریزی و موادرسانی به ترتیب در حدود 6/97، 95، 7/89 و 83 دسی‌بلA و تراز صوت در سه زیرواحد کوره بلند، سالن بلوور و سالن کولینگ تاور بیش از 90 دسی‌بل برآورد گردید. میانگین دز صدای دریافتی كارگران در زیرواحدهای کوره، چدن‌ریزی، بلوور، بگ فیلتر و کولینگ تاور به ترتیب8/282%، 12/295%، 9/239% 234% و 5/180% تعیین گردید. میانگین افت شنوایی ناشی از صدا در طول سه سال متوالی به ترتیب 4/12، 5/14 و 4/15 دسی‌بل A بود. نتیجه‌گیری: واحدهای کوره، انرژی و چدن‌ریزی دارای شرایط نامناسبی به لحاظ آلودگی صوتی می‌باشند به‌طوری‌که میانگین افت شنوایی کارگران آن‌ها در هر سال 5/1 دسی‌بل افزایش داشته است. لذا باید اقدامات کنترلی مناسبی در این واحدها صورت گیرد.

Title: بررسی وضعیت استرس شغلی و رابطه آن با تعهد سازمانی و عملکرد شغلی در بین کارکنان سازمان زندان های استان ایلام

http://hsr.mui.ac.ir/index.php/jhsr/article/view/2054

مقدمه: استرس شغلی می‌تواند باعث ایجاد تأثیرات منفی بر روی متغیرهای سازمانی مانند تعهد و وفاداری افراد به سازمان و متغیرهای فردی سازمانی مانند عملکرد شغلی افراد گردد. هدف از انجام این مطالعه بررسی وضعیت استرس شغلی و تأثیر آن بر روی تعهد سازمانی و عملکرد شغلی در کارکنان سازمان زندان‌های استان ایلام بود. روش‌ها: این مطالعه بر روی 177 نفر از کارکنان سازمان زندان‌های استان ایلام و در زندان مرکزی ایلام، مجتمع حرفه آموزی و کاردرمانی دالاب، زندان دره شهر و کانون اصلاح و تربیت انجام گرفت. به منظور جمع آوری اطلاعات از مقیاس استرس شغلی سازمان اجرایی بهداشت و ایمنی انگلستان (HSE یا Health and Safety Executive) ، پرسش‌نامه تعهد سازمانی آلن و مایر (Allen & Meyer's Organizational Commitment Questionnaire) و پرسش‌نامه عملکرد شغلی پاترسون (Job Performance Questionnaire Paterson) استفاده گردید. داده‌ها با استفاده از نرم افزار SPSS نسخه 20 و آزمون‌های آماری چند متغیره، مدل خطی عمومی، ضریب همبستگی پیرسون و ANOVA تجزیه و تحلیل شدند. یافته‌ها: نتایج نشان داد که میانگین نمره استرس شغلی (± انحراف معیار) در زندان مرکزی ایلام، مجتمع حرفه آموزی و کاردرمانی دالاب، زندان دره شهر و کانون اصلاح و تربیت، به ترتیب 74/98 (81/14±)، 60/95 (08/15±)، 12/99 (12/19±) و 71/85 (71/15±) بود. هم‌چنین مشخص شد که بین استرس شغلی و تعهد سازمانی ارتباط معنی‌داری وجود نداشت، اما بین استرس شغلی و عملکرد شغلی افراد از نظر آماری رابطه معنی‌داری وجود داشت (000/0P=). نتیجه‌گیری: استرس شغلی بر روی تعهد سازمانی کارکنان زندان اثری نداشت اما بر روی عملکرد شغلی آن‌ها تأثیرگذار بود و باعث کاهش عملکرد آن‌ها شد. به کارگیری اقدامات لازم جهت مدیریت، کاهش یا حذف استرس شغلی و استرسورهای مرتبط با آن در جهت حفظ سلامت فردی و شغلی کارکنان زندان و افزایش کیفیت و کمیت عملکرد شغلی آنها، امر ضروری می‌باشد.

Title: مقایسه‌ی روش های ممیزی کلاسیک با شبکه ی عصبی مصنوعی به وسیله چند الگوریتم مختلف در تشخیص بیماری انفارکتوس میوکارد

http://hsr.mui.ac.ir/index.php/jhsr/article/view/2160

مقدمه: تحلیل ممیزی و رده‌بندی یکی از پرکاربردترین بخش‌های آماری در حوزه های مختلف علمی است. در این مورد روش‌های کلاسیک آماری دارای پیش‌فرض‌هایی هستند که در صورت برقرار نبودن آن‌ها استفاده از این روش‌ها با خطاهای قابل توجه‌ای همراه است. از طرفی روش شبکه عصبی مصنوعی با محدودیت کمتری روبرو است، و از آن‌جا که تحلیل داده‌های پزشکی دارای حساسیت بالایی است و اشتباه در رده‌بندی منجر به خطاهای جبران‌ناپذیری خواهد شد اهمیت دارد که دقیق‌ترین روش با کم‌ترین خطا را برای تحلیل این داده‌ها به کار برد. روش‌ها: برای مقایسه‌ی سه روش ممیزی درجه دو، رگرسیونلجستیک و شبکه عصبی مصنوعی در تشخیص بیماری از 1000 داده مربوط به بیماری انفارکتوس میوکارد استفاده شد. ارزیابی شبکه عصبی، با ملاک حداقل مربعات خطای پیش‌بینی صورت پذیرفت و از الگوریتم پس‌انتشار خطا استفاده شد. در ادامه عملکرد سه الگوریتم مختلف (BFGS یا Broyden-Fletcher-Goldfarb-Shanne) , Conjugate gradient و Gradient descent از شبکه عصبی در تشخیص و پیش‌بینی بیماری، مورد مقایسه قرار گرفت. تحلیل داده‌ها با نرم افزارهایSPSS, R, SAS, STATISTICA انجام شد. یافته‌ها: درصد خطا، درصد صحت پیش‌بینی، حساسیت، ویژگی و سطح زیر منحنی راک به ترتیب در روش ممیزی درجه دو برابر با 15/10، 85/89، 8888/0، 9083/0 ، 922/0 و در روش رگرسیون لجستیک برابر با 88/10، 12/89، 8743/0، 9110/0، 941/0 و برای شبکه عصبی مصنوعی منتخب برابر با 97/3، 03/96، 9561/0، 9644/0، 966/0 به دست آمد. تفاوت معنی‌داری بین سطح زیر منحنی راک برای سه روش وجود داشت. هم‌چنین از بین سه الگوریتم مختلف شبکه عصبی الگوریتم BFGS بر اساس معیارهای ذکر شده عملکرد بهتری داشته است و تفاوت معنی‌داری بین سطح زیر منحنی راک برای سه الگوریتم وجود داشت. نتیجه‌گیری: با توجه به این‌که محدودیتی برای استفاده از شبکه عصبی مصنوعی وجود ندارد می‌توان نتیجه گرفت که این روش دقت پیش‌بینی و غربالگری بهتری نسبت به روش‌های ممیزی لجستیک و ممیزی درجه دو در تشخیص و پیش‌بینی بیماری انفارکتوس میوکارد داشته است.

Title: مدیریت و بهینه‌سازی میزان تزریق کلر در شبکه‌های توزیع آب با استفاده از مدل Water Gems v3/0

http://hsr.mui.ac.ir/index.php/jhsr/article/view/1649

مقدمه: آب آشاميدني به روش‌هاي مختلفي گندزدايي مي‌شود كه متداول‌ترين و ارزان‌ترين روش آن، کلرزني است. يکي از مهم‌ترين مسائل در زمينه کلرزنی،  مکان و ميزان تزريق کلر است که بايد به گونه‌اي انتخاب شود که ميزان کلر باقيمانده در کليه نقاط يک شبکه توزيع آب شهري در محدوده استاندارد بوده و هم‌چنين هزينه‌هاي مربوطه حداقل گردد. در این تحقیق سعی شده با اجرای برنامه برای دو شبکه توزیع آب شهری، میزان کلر باقیمانده در لوله‌ها و مقدار کلر مصرفی در نقاط مختلف شبکه مورد مقایسه و تحلیل کیفی قرار گیرد. روش‌ها: به‌منظور مدل‌سازی شبکه از نرم‌افزار  water Gems v3/0 استفاده شد. با استفاده از این نرم‌افزار، شبکه به دو صورت ثقلی و پمپاژ مستقیم، مدل و طراحی گردید. یافته‌ها: با حل دو مدل كاربردي از شبكه‌هاي آبرساني توسط نرم‌افزار، کارايي هر دو شبکه در جهت تعيين ميزان بهينه تزريق کلر نشان داده شد. نتایج نشان داد که میزان کلر باقی‌مانده در 62٪ از لوله‌های شبکه پمپاژ مستقیم در حد استاندارد و در 100٪ از لوله‌های موجود در شبکه توزیع ثقلی کمتر از حد استاندارد است. هم‌چنین میزان کلر مصرفی در شبکه پمپاژ مستقیم 5/6٪ کمتر از شبکه توزیع ثقلی محاسبه گردید. نتیجه‌گیری: این تحقیق نشان داد که بهینه‌سازی تزریق کلر به شبکه‌های با پمپاژ مستقیم در مقایسه با شبکه‌های توزیع ثقلی، بیشتر امکان‌پذیر بوده و هم‌چنین به میزان کلر مصرفی کمتری نیاز می‌باشد.

Title: ارتباط بین از دست‌دهی دندان و سندرم روده تحریک‌پذیر در بزرگسالان ایرانی

http://hsr.mui.ac.ir/index.php/jhsr/article/view/1890

مقدمه: هر چند ارتباط بین تعداد دندان‌ها و اختلالات گوارشی شناخته شده است، اطلاعات محدودی در مورد رابطه بین از دست دادن دندان‌ها و سندرم روده تحریک‌پذیر (IBS یا Irritable Bowel Syndrome) موجود است. هدف از این مطالعه بررسی ارتباط بین وضعیت دندانی و IBS در بین بزرگسالان ایرانی بود. روش‌ها: در این مطالعه مقطعی بر روی 4012 بزرگسال ایرانی، وضعیت دندانی به وسیله پرسش‌نامه خود اجرا ارزیابی شد. شرکت‌کننده‌ها در سه گروه اصلی طبقه‌بندی شدند: افراد دارای کلیه دندان‌ها، 5-1 دندان از دست داده، بیش از 5 دندان از دست داده‌اند. IBS و زیر گروه‌های آن بر اساس معیارهایRome IIIتعریف شدند. یافته‌ها: پس از تعدیل متغیرهای مخدوشگر، افرادی که 5-1 دندان را از دست داده بودند، 33/1 برابر شانس بیشتری برای ابتلا به IBS در مقایسه با افراد دارای کلیه دندان‌ها، داشتند. پس از کنترل متغیرهای مخدوشگر افرادی که 5-1 دندان را از دست داده بودند، از 37٪ شانس بیشتری برای ابتلا به IBS با غلبه یبوست، در مقایسه با افراد دارای کلیه دندان‌ها برخوردار بودند (Odds Ratio: 37/1 ٪؛ 95٪ فاصله اطمینان: 86/1-01/1). چه در مدل خام و چه در مدل‌های تعدیل شده، رابطه معنی‌داری بین وضعیت دندانی و سایر زیرگروه‌های IBS دیده نشد. هم‌چنین رابطه‌ای بین از دست دادن بیش از 5 دندان و شیوع IBS یافت نشد. نتیجه‌گیری: مطالعه حاضر نشان داد از دست دادن 5-1 دندان به صورت معنی‌داری خطر IBS را افزایش می‌دهد به خصوص در مورد IBS با غلبه یبوست. این رابطه در گروه مردان برخلاف زنان معنی‌دار بود. جهت تأیید این یافته‌ها نیاز به مطالعات بیشتر از نوع آینده‌نگر می‌باشد.

Title: بررسی کارایی کربن فعال اصلاح شده با نانو ذرات اکسید روی در حذف رنگ متیلن بلو از فاضلاب سنتتیک: مطالعه کینتیک و ایزوترم

http://hsr.mui.ac.ir/index.php/jhsr/article/view/2026

مقدمه: : پساب‌های حاوی رنگ‌های سنتتیک برای سیستم‌های اکولوژیکی و سلامت عمومی خطرناک هستند. فرایند جذب سطحی در سال‌های اخیر توجهات زیادی را به خود جلب کرده است. لذا این مطالعه با هدف بررسی امکان استفاده از نانو ذرات اکسید روی تثبیت شده بر کربن فعال در جذب رنگ متیلن بلو از فاضلاب سنتتیک انجام شد. روش‌ها: مطالعه حاضر به صورت آزمایشگاهی در یک راکتور منقطع انجام گرفت. تثبیت کردن نانوذرات بر روی کربن فعال به روش حرارتی صورت گرفت. اثر متغیرهایی نظیر بارگذاری نانوذرات بر سطح کربن فعال، pH، زمان تماس، غلظت رنگ و دوز جاذب مطالعه شد.کینتیک واکنش و ایزوترم جذب مورد بررسی قرار گرفت. یافته‌ها: مقادیر ظرفیت جذب رنگ در بارگذاری‌های نانوذرات 01/0، 1/0 و 5/0گرم بر یک گرم کربن فعال برای غلظت اولیه رنگ 50 میلی‌گرم در لیتر و جرم جاذب 3/0 گرم در لیتر در زمان 120 دقیقه به ترتیب 6/16، 45/16 و 52/14 میلی گرم بر گرم بوده است. میزان جذب رنگ با جاذب پوشش داده شده (با ظرفیت جذب 6/16میلی‌گرم بر گرم) از قدرت رنگبری بالاتری نسبت به کربن خام (با ظرفیت جذب 47/6 میلی گرم بر گرم) برخوردار است. نتیجه‌گیری: نتایج حاصل مشخص ساخت که می‌توان از فن‌آوری تثبیت نانوذرات بر کربن فعال به عنوان یک جاذب مؤثر، کارآمد و سریع در جهت حذف رنگ متیلن بلو از محلول‌های آبی استفاده کرد.

Title: کاربرد کربن فعال شده با کلرید آمونیوم (NAC) در حذف کروم شش ظرفیتی از آب‌های آلوده: بررسی سینتیک و ایزوترم جذب

http://hsr.mui.ac.ir/index.php/jhsr/article/view/2217

مقدمه: کروم از فلزات سنگین موجود در پساب صنایع بوده که به‎ دلیل اثر منفی بر موجودات زنده و اثرات زیست محیطی باید قبل از تخلیه به محیط، از آب‎های آلوده حذف گردد. هدف از این مطالعه، بررسی پتانسیل جذب کربن فعال شده با کلرید آمونیوم برای حذف کروم شش ظرفیتی از محلول‌های آبی و مقایسه آن با کربن فعال تجاری می‎باشد. روش‌ها: در این مطالعه، اثر pH، غلظت اولیه کروم شش ظرفیتی، مقدار جاذب، زمان تماس بر راندمان حذف کروم بررسی شد. همچنین ایزوترم و سینتیک جذب کروم توسط کربن فعال شده با کلرید آمونیوم (NAC) و کربن فعال تجاری (SAC) مورد بررسی قرار گرفت . یافته‌ها: نتایج تحقیق نشان داد که کربن فعال شده با کلرید آمونیوم (NAC) می‌تواند در غلظت کرومmg/l  50، دوز بهینه 1 گرم در لیتر، pH 2، و زمان تماس 30 دقیقه بیش از 90 در صد کروم را حذف نماید. فرایند جذب از مدل سینتیک شبه درجه دوم نوع 1 تبعیت کرده و داده‌‌های جذب با ایزوترم لانگمویر نوع 1 مطابقت بیشتری داشت. حداکثر ظرفیت جذب برای NAC و SAC به ترتیب  mg/g85/142 و mg/g 69/63 به دست آمد. نتیجه‌گیری: نتایج نشان داد که NAC در مقایسه با SAC به عنوان یک جاذب موثر و مقرون به صرفه در حذف کروم شش ظرفیتی از آب‌های آلوده می‌باشد.

Title: کاربرد روش استخراج فاز جامد بر پايه نانوزئوليت Y براي سنجش مقادير جزيي فلزات سنگین كادميم Cd2+)) و كروم ) Cr6+ ( ازمحلول‌های آبی

http://hsr.mui.ac.ir/index.php/jhsr/article/view/2265

مقدمه: مواجهه با فلزات سنگین به عنوان یک خطر بهداشتی و زیست محیطی مطرح مي‌باشد. سنجش عناصر در محلول‌هاي مختلف، مقدمه‌اي برای پيشگيري وكنترل مواجهه با فلزات است. امروزه روش‌هاي آماده‌سازی نمونه، براي تسهيل شيوه‌هاي آناليز استفاده مي‌شود. در این مطالعه، کاربرد روش استخراج فاز جامدي بر پايه نانوزئوليت  Yبراي تغليظ مقادير بسیار کم Cd2+و Cr6+  از محلول‌های آبی مورد بررسی قرار گرفته است. روش‌ها: در این مطالعه با كپسوله كردن ليگاندي درون ميسل‌هاي تريتون X-100 و هدايت اين مجموعه درون حفرات نانوزئوليت Y، فاز جامد تهيه شد. فاز جامد تهيه شده با pH هایي در رنج 5/2 تا 5، در غلظت‌هاي مختلف ليگاند و زمان به هم خوردن10 تا 80 دقیقه بررسي گرديد. استخراج فلزات با مقادير بسيار كم، توسط فاز جامد و به كمك دستگاه جذب اتمي كوره، ارزيابي و اعتباربخشي گرديد. یافته‌ها: : منحني استاندارد براي هر دو فلز بعد از استخراج در رنج غلظت 5 تا 80 ميكروگرم بر ليتر(ppb)، با ضريب همبستگي بيش از 99% به دست آمد. ضريب تغييرات (Coefficient variance) درون روز و برون روز براي استخراج فلزات كروم (VI) وكادميم(II) از فاز جامد بهينه شده در غلظت‌هاي مختلف كمتر از 7٪ گزارش گرديد. نتیجه‌گیری: نتايج مطالعه نشان مي‌دهد با مهار كردن شلاته كننده‌اي مثل آمونيم پيروليدين دي تيو كاربامات درون ميسل‌هايي از تريتون X-100  مي‌توان تركيبي ايجاد نمود كه فلزات در مقادير بسيار كم را به دام اندازد. بررسي فاكتورهاي مختلف مثل تكرارپذيري و صحت روش استخراج مشخص ساخت كه  نانوزئوليت Y عامل‌دار شده در استخراج  Cd2+و Cr6+ ازمحلول‌های آبی، موفقيت‌آميز عمل كرده است. پیشنهاد می‌شود جهت تعیین مقدار فلزات یاد شده از محلول‌های آبی فاز جامد بهینه شده استفاده گردد.

Title: رویکرد نیمه‌پارامتری بیزی به خوشه‌بندی رگرسیونی کودکان و نوجوانان ایرانی بر اساس ریسک ابتلا به بیماری‌های قلبی- عروقی و دیابت

http://hsr.mui.ac.ir/index.php/jhsr/article/view/2497

مقدمه: در این مقاله، خوشه‌بندی رگرسیونی(Regression clustering)  با استفاده از فرایند دیریکله (Dirichlet process) جهت به‌دست آوردن بینشی جامع از الگوی سلامت کودکان و نوجوانان بررسی‌شده در مطالعات ملی ایران، درنظر گرفته شد. در این رویکرد به خوشه‌بندی، ضمن این‌که تأثیر عوامل مزاحم، حذف و تحلیل‌های دقیق‌تری از وضعیت افراد به‌دست می‌آید، تعداد خوشه‌ها و الگوهای موجود در داده‌ها نیز تخمین زده می‌شود. در این پژوهش، خوشه‌بندی افراد نمونه از نظر دو شاخص چربی‌خون و قند خون، مدنظر قرار گرفته و میزان تأثیر شاخص تن‌سنجی (Anthropometric)، رده سنی و جنسیت بر نحوه خوشه‌بندی افراد، مورد ارزیابی قرار گرفته است. روش‌ها: به منظور برآورد پارامترهای مجهول مدل، با رویکرد بیز (Bayesian approach) به مسأله، از روش‌های شبیه‌سازی مونت کارلوی زنجیر مارکفی (Markov chain Monte Carlo) در نرم‌افزار اپن‌باگز (Open Bugs) استفاده شده است. به‌منظور به‌دست آوردن بینش مناسب در رابطه با تعداد الگوهای افراد در معرض خطر بیماری‌های قلبی- عروقی و دیابت، بر مبنای شاخص چربی‌خون و قند خون، از فرایند دیریکله استفاده شده است. یافته‌ها: تأثیر متغیرهای شاخص تن‌سنجی، رده سنی و جنسیت بر خوشه‌بندی با وارد کردن آن‌ها به‌عنوان متغیرهای توضیحی در مدل رگرسیونی و درنتیجه حذف اثر آن‌ها بر خوشه‌بندی، مورد بررسی قرار گرفت. نتایج منجر به تشکیل سه خوشه شد، به‌طوری که درصد افراد تخصیص‌یافته به خوشه‌های 1 تا 3، به‌ترتیب برابر با 47% (1310 نفر)، 4%(112 نفر) و 49%(1366نفر) شد. با بررسی چارک‌های متغیرهای قند خون و چربی‌خون، نتیجه‌ می‌شود که افراد قرارگرفته در خوشه 3 که حجم بزرگی از کودکان و نوجوانان نمونه را تشکیل می‌دهند، از لحاظ شاخص چربی‌خون و نیز قند خون، افراد در معرض خطر محسوب می‌شوند. هم‌چنین افراد خوشه اول دارای قند خون و شاخص چربی خون نرمال و افراد خوشه دوم دارای قند خون در محدوده خطر و شاخص چربی خون نرمال هستند. نتیجه‌گیری: در خوشه‌بندی کودکان و نوجوانان از لحاظ دو متغیر قند خون و شاخص چربی‌خون، متغیرهای جنسیت، رده سنی و شاخص تن‌سنجی عوامل تأثیرگذار بر تعداد و ساختار خوشه‌ها هستند. هم‌چنین، افراد نمونه به سه خوشه، افراد با وضعیت نرمال، افراد در ریسک ابتلا به بیماری‌ دیابت و افراد در ریسک بیماری‌های قلبی- عروقی و نیز دیابت، تقسیم‌بندی شدند.

Title: بررسی اثربرنامه های ترویجی-آموزشی بر میزان کاهش مصرف غذای آماده (مطالعه موردی : ارائه کلیپ ،بروشور و مشاوره به دانش آموزان دبستان سما)

http://hsr.mui.ac.ir/index.php/jhsr/article/view/2455

مقدمه: بازاریابی اجتماعی در طی چندین سال گذشته بارها در جهت تأثیرگذاری بررفتار بهداشتی مورد استفاده قرارگرفته شده است. هدف این مطالعه بررسی اثر ارائه برنامه‌های ترویجی-آموزشی که شامل بروشور، کلیپ‌های تبلیغاتی و مشاوره می‌باشد بر کاهش مصرف غذای آماده دانش آموزان دبستان سما می‌باشد. روش‌ها: این مطالعه نیمه تجربی بر روی 56 نفر از دانش‌آموزان دبستان دخترانه و پسرانه سما در دو گروه آزمایش و کنترل انجام شد. ابزار گردآوري اطلاعات پرسش‌نامه‌اي شامل دو قسمت بود سؤالات زمینه‌ای و سؤالاتی در مورد میزان مصرف غذای آماده که با توجه به مطالعات قبلی طراحی شد. این پرسش‌نامه به صورت پیش‌آزمون در هر دو گروه تکمیل شد. سپس بر اساس اطلاعات به دست آمده برنامه ترویجی- آموزشی که شامل کلیپ تبلیغاتی و بروشور و مشاوره حضوری بود برای گروه آزمایش طراحی و اجرا شد و پس از سه هفته آزمون ثانویه از هر دو گروه به عمل آمد. بعد از جمع‌آوری داده‌ها با استفاده از آزمون فرضیه‌های مختلف از جمله تحلیل واریانس فرضیه‌ها مورد تجزیه و تحلیل قرار گرفتند. یافته‌ها: میانگین نمره برنامه‌های ترویجی آموزشی قبل از آموزش در 2 گروه اختلاف معنی‌داری نداشت (39/0p=). اما بعد از آموزش میانگین نمره برنامه‌های ترویجی آموزشی در گروه آزمایش نسبت به گروه کنترل اختلاف معنی‌داری داشت  (002/0p=). نتایج نشان دادند که بعد از آموزش بین ارائه کلیپ  (000/0p=) ، ارائه بروشور (000/0p=) و دادن مشاوره (000/0p=) بر کاهش مصرف غذای آماده در گروه آزمایش ارتباط معنی‌داری وجود دارد. نتیجه‌گیری: برنامه‌های ترویجی آموزشی در مورد کاهش مصرف غذای آماده در رفتار تغذيه‌اي دانش‌آموزان تحت مطالعه تأثير مثبت داشته است. با توجه به پايين بودن سطح آگاهي دانش‌آموزان مقطع ابتدایی و براي بهبود و اصلاح رفتار تغذیه‌اي آنان در مورد کاهش مصرف غذای آماده استفاده از برنامه ترویجی آموزشي ضروري به نظر مي‌رسد.
